# Human papillomavirus vaccination coverage in Northeast Brazil, 2013-2021: a descriptive study

**DOI:** 10.1590/S2237-96222023000200012

**Published:** 2023-05-19

**Authors:** Mateus de Paula von Glehn, Luciana Maiara Diogo Nascimento, Krishna Mara Rodrigues Freire, Thaís Tâmara Castro e Souza Minuzzi, Carlos Edson Hott, Ana Goretti Kalume Maranhão, Camile de Moraes

**Affiliations:** 1 Ministério da Saúde, Secretaria de Vigilância em Saúde e Ambiente, Brasília, DF, Brazil

**Keywords:** Immunization Programs, Adolescent Health, Vaccination Coverage, Health Information Systems, Papillomavirus vaccines, Descriptive Epidemiology, Programas de Inmunización, Salud del Adolescente, Cobertura de Vacunación, Sistemas de Información en Salud, Vacunas contra Papillomavirus, Epidemiología Descriptiva, Programas de Imunização, Saúde do Adolescente, Cobertura Vacinal, Sistemas de Informação em Saúde, Vacinas contra Papilomavírus, Epidemiologia Descritiva

## Abstract

**Objective:**

to describe human papillomavirus (HPV) vaccination coverage in the Northeast region of Brazil, in the period from 2013 to 2021.

**Methods:**

this was a descriptive study conducted with data obtained from the National Immunization Program, which sets a goal of 80% coverage of HPV vaccination in girls aged between 9 and 14 years and boys aged between 11 and 14 years.

**Results:**

HPV vaccination coverage in girls was 73.9%, regarding the first dose, and 54.3% regarding the second dose, and for boys, the coverage of each dose was 49.7% and 32.6%, respectively; with the exception of the states of Ceará and Paraíba, which reached coverage above 80% regarding the first dose in girls, none of the states reached the goal for both doses.

**Conclusions:**

between 2013 and 2021, HPV vaccination coverage was below the target for both sexes, with the exception of the states of Ceará and Paraíba, which reached the goal for the first dose in the girls.

## Resumo

**Objetivo:**

descrever a cobertura da vacina contra papilomavírus humano (HPV) na região Nordeste do Brasil, no período de 2013 a 2021.

**Métodos:**

estudo descritivo conduzido com dados obtidos do Programa Nacional de Imunizações, que estabelece a meta de 80% para a vacina contra o HPV para meninas entre 9 e 14 anos e meninos entre 11 e 14 anos.

**Resultados:**

as coberturas para as meninas foram de 73,9%, na primeira, e de 54,3% na segunda dose, e para meninos, as coberturas de cada dose foram de 49,7% e 32,6%, respectivamente; excetuando-se Ceará e Paraíba, que alcançaram coberturas acima de 80% na primeira dose para as meninas, nenhum estado alcançou a meta para as duas doses.

**Conclusões:**

entre 2013 e 2021, as coberturas da vacina contra HPV estiveram abaixo da meta para ambos os sexos, com exceção de Ceará e Paraíba, que atingiram a meta para a primeira dose no grupo de meninas.

## Resumen

**Objetivo:**

describir las coberturas de la vacuna contra el papilomavirus humano en la Región Nordeste de Brasil y sus estados, de 2013 a 2021.

**Métodos:**

se trata de un estudio descriptivo realizado con datos de cobertura vacunal obtenidos del Programa Nacional de Immunizaciones, que establece la meta del 80% para la vacuna. Los datos de población se obtuvieron del Departamento de Informática del Ministerio de Salud.

**Resultados:**

la cobertura de vacunación en niñas fue del 73,9% en la primera y del 54,3% en la segunda dosis; en niños la cobertura de cada dosis fue del 49,7% y 32,6%; Ceará y Paraíba alcanzaron una cobertura superior al 80% para la primera dosis en niñas, y ningún estado alcanzó la meta para las dos dosis.

**Conclusiones:**

la cobertura de la vacuna está por debajo de la meta para ambos sexos, con excepción de la primera dosis en niñas en Ceará y Paraíba.

**Table:** 

Study contributions	
**Main results**	In the period from 2013 to 2021, human papillomavirus (HPV) vaccination coverage for adolescents in the Northeast region of Brazil was higher for the first dose, in both sexes.
**Implications for services**	The qualification and monitoring of HPV immunization data are strategic actions to increase vaccination coverage, and to guiding local actions aimed at vaccination for adolescents in schools.
**Perspectives**	Managers can improve strategies to increase vaccination coverage, including community mobilization. Vaccination coverage surveys can contribute to a better understanding of the situation at the local level.

## INTRODUCTION

Human papillomavirus (HPV) is a group of more than 200 viruses,^1^ at least 13 of them are considered oncogenic.^2^ Genotypes 16, 18, 45, 31, 33, 35, 39, 45, 51, 52, 58, 59, and 68 are associated with persistence of infection and development of cancer, such as cervical and penile cancers. HPV genotypes 16 and 18 are found in up to 70% of cervical cancer cases and, although they are considered low-risk HPV types, genotypes 6 and 11 are associated with about 90% of genital warts.^3^

A cohort study conducted with data from 2004-2014 decade showed that Maranhão, one of the states in the Northeast region of Brazil, has the highest incidence of penile cancer worldwide,^4^ and a recent meta-analysis, which investigated the detection and genotypic distribution of HPV in cases of penile cancer in Brazil, pointed out that the highest prevalence of HPV in these cases occurs in the Northeast region (79%), with HPV16 being the most frequently detected, followed by HPV6, both with protection conferred by the HPV vaccine.^5^

HPV infection is the most common of all sexually transmitted infections, and its prevalence varies among different populations and age groups.^6^ In Brazil, a cross-sectional study conducted in the 27 Federative Units (FUs) between 2016 and 2017 found that the prevalence of this infection among young people aged 16 to 25 years was 53.6%, with a predominance in the Northeast region (53.1%).^7^

In 2020, there were 6,627 deaths due to cervical cancer in Brazil, of which 2,058 (31%) occurred in the Northeast region,^8^ thus, the region was ranked second in absolute number of deaths and mortality coefficient from the disease, behind only the North region. It is noteworthy that cervical cancer is a preventable disease and that HPV vaccination, along with screening for the disease, are the main strategies for reducing cases and deaths due to this condition.^9^

The HPV vaccine was introduced into the national immunization schedule in 2014,^10^ preceded by initiatives in the state of Amazonas and in the Federal District.^11^ Initially the recommended schedule included a three-dose regimen administered at intervals of 0, 6 and 60 months for girls aged 11 to 13 years, with gradual expansion to other age groups, until the indication for girls in the age group from 9 to 14 years, in 2016, and for boys aged 11 to 14 years, in 2017.^12^ However, the recommended vaccination schedule changed in 2016 to two doses, with an interval of six months between them, following recommendations from the World Health Organization (WHO).^13^

Taking into consideration the prevalence of HPV infection and the associated complications reported in the Northeast region, the objective of this study was to describe the HPV vaccination coverage in Northeastern Brazil, from 2013 to 2021.

## METHODS

### Study design

This was a descriptive study, conducted with secondary data extracted from the National Immunization Program Information System (*Sistema de Informação do Programa Nacional de Imunizações* - SIPNI).

### Setting

The doses of the HPV vaccine administered are recorded on information systems made available by the Ministry of Health, such as the e-SUS Primary Care Systems (*e-SUS Atenção Primária* e-SUS APS) and SIPNI. Vaccine dose records can also be made in public or private systems. All vaccine dose records should be entered or integrated into information systems made available by the Ministry of Health, so that it is possible to monitor vaccination by FU, municipality, sex and age group.^14^

System integration allows the elaboration of anonymized reports, with the indicators of doses administered, vaccination coverage and homogeneity of coverage, which are extracted from the statistical tabulation application called TABNET, developed by the Ministry of Health and made available in the Brazilian National Health System Information Technology Department (*Departamento de Informática do Sistema Único de Saúde* - DATASUS - http://tabnet.datasus.gov.br). In these reports, vaccination indicators are presented in aggregate form (anonymized) by municipality, FU, region and country.^14^

### Participants

Cohorts from 9 to 15 years of age for the female population and 11 to 15 years old for the vaccinated male population in the Northeast region were defined. The second dose of the vaccine was evaluated in the population aged up to 15 years of both sexes, according to the cohort event monitoring strategy proposed by the WHO and the Pan American Health Organization (PAHO), followed by the National Immunization Program (*Programa Nacional de Imunizações* - PNI).^13^ ^,^ ^15^ The calculation of vaccination coverage in 2021 was performed as follows:

1. 9-year-old population: population vaccinated at age 9 in 2021;
2. 10-year-old population: population vaccinated at age 9 in 2020, added to the vaccinated population aged 10 years in 2021;
3. 11-year-old population: sum of populations vaccinated at age 9 in 2019; at age 10 in 2020; and at age 11 in 2021;
4. 12-year-old population: sum of populations vaccinated at age 9 in 2018; at age 10 in 2019; at age 11 in 2020; and at age 12 in 2021.

This procedure was performed successively for the population vaccinated at 13, 14 and 15 years of age. Regarding the calculation of the doses administered, the first and second doses for females and males were considered separately.

### Variables

The study variables were as follows: sex (male or female), age (9 to 15 years old, for girls, and 11 to 15 years old, for boys); dose administered (first or second); vaccination coverage (< 50%, between 50% and 80%, and greater than or equal to 80%); and homogeneity of vaccination coverage among municipalities (number of municipalities in the state with vaccination coverage above 80% divided by the total number of municipalities in the state).

### Data sources and measurement

The data were extracted on August 11, 2022, using the TabNet tabulation application. In order to calculate vaccination coverage, the number of doses administered as of 2014 was considered as numerator. For the denominator, we used the population comprised of girls and boys at each age, estimated by the Brazilian Institute of Geography and Statistics (*Instituto Brasileiro de Geografia e Estatística* - IBGE) in 2010 and updated in 2021 by the General Coordination of Epidemiological Information and Analysis of the Department of Health Analysis and Surveillance of Noncommunicable Diseases of the Ministry of Health.^16^

Homogeneity was calculated by dividing the number of municipalities in the state with vaccination coverage above 80% (numerator) by the total number of municipalities in the state (denominator). The Northeast region has a total of 1,794 municipalities, distributed over its nine states: Alagoas (102 municipalities), Bahia (417 municipalities), Ceará (184 municipalities), Maranhão (217 municipalities), Paraíba (223 municipalities), Pernambuco (185 municipalities), Piauí (224 municipalities), Rio Grande do Norte (167 municipalities) and Sergipe (75 municipalities).^8^ ^,^ ^14^

### Study size

All doses administered between 2013 and 2021 to both sexes, were taken into consideration.

### Statistical methods

As of 2016, the Ministry of Health adopted the calculation by age cohorts as a methodology for evaluating HPV vaccination coverage.^14^ This type of calculation takes into account the cumulative doses, since the year of vaccine implementation for each cohort, taking into consideration that this is a two-dose regimen, which can be administered in different years. The calculation of coverage was performed in a similar way for both the first dose and the second dose, and it was compared to the goal set by the PNI (80%).^14^ The distribution (absolute and relative frequencies) of vaccination coverage by municipality in each state was described.

Data were presented in relative and absolute frequencies and organized in graphs and maps, consolidated by municipality and state, using Microsoft Office Excel 2016 and QGIS version 3.16.1.^8^ The latter is a free open-source cross-platform desktop Geographic Information System (GIS) application, which supports viewing, editing and analysis of geospatial data. 

### Ethical aspects

The study was exempted from the approval of a Research Ethics Committee (REC), given that it is an evaluation of secondary data in the public domain (National Health Council Resolution No. 674, of May 6, 2022).

## RESULTS

**Figure 1:**
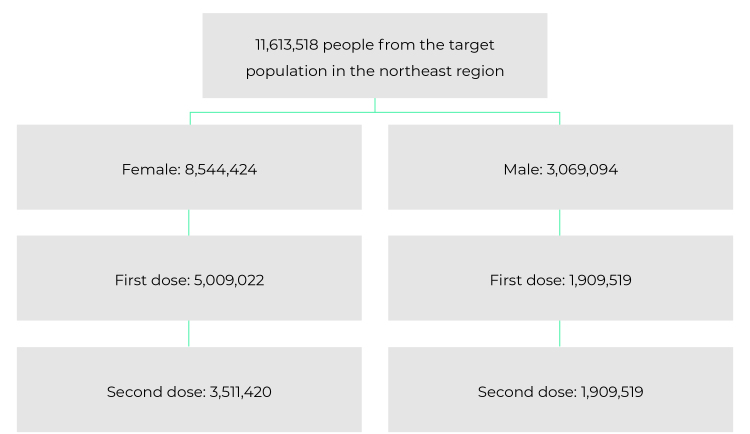
- Distribution of the number of doses of human papillomavirus (HPV) vaccine, administered by sex and according to the dose (first or second) of the vaccination schedule, in the Northeast region, Brazil, 2013-2021

In the period evaluated, 11,613,518 doses of HPV vaccine were administered in the Northeast region of Brazil, of which 8,544,424 doses were for females (5,009,022 for the first dose and 3,511,420 for the second dose) and 3,069,094 for males (1,909,519 for the first dose and 1,150,372 for the second dose) (Figure 1).

**Figure 2:**
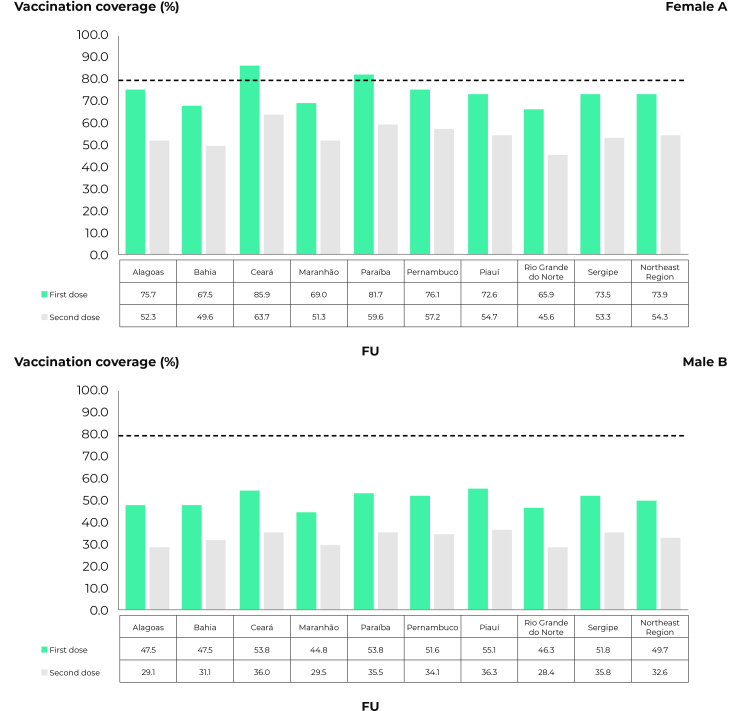
- Distribution of human papillomavirus (HPV) vaccination coverage in female (A) and male (B) populations, by state, in the Northeast region, Brazil, 2013-2021 (N = 11,613,518)

As for females, vaccination coverage in the region was 73.9% regarding the first dose and 54.3% regarding the second dose. The states with the highest coverage were Ceará (85.9%) and Paraíba (81.7%), both for the first dose and the second dose, while the lowest coverage was found in the state of Rio Grande do Norte (65.9% regarding the first dose and 45.6% regarding the second dose) (Figure 2A).

With regard to the male population, the coverage of the first dose in the region was 49.7%, while the coverage of the second dose was 32.6%. In relation to this , the states with the highest coverage were Ceará (53.8%) and Paraíba (53.8%), for the first dose, and Piauí (36.3%) and Ceará (36.0%), for the second dose. The lowest coverage was found in the state of Maranhão, for the first dose (44.8%), and in Rio Grande do Norte, for the second dose (28.4%) (Figure 2B).

**Figure 3:**
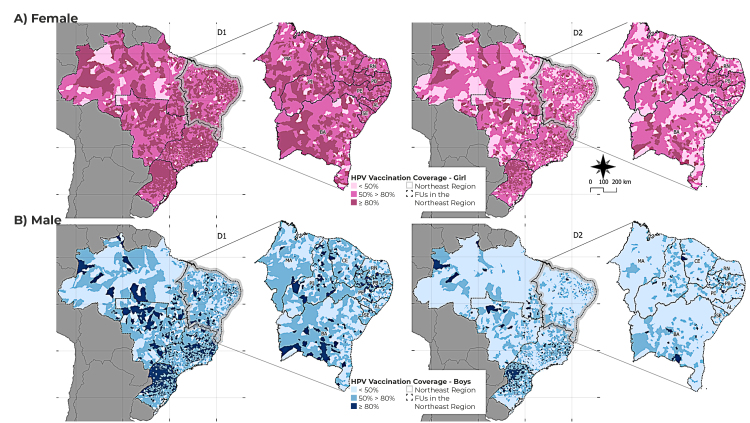
- Spatial distribution of human papillomavirus (HPV) vaccination coverage in females (A) and males (B) in the Northeast region, Brazil, 2013-2021 (N = 11,613,518)

When evaluating spatially, it could be seen that 50.6% (908/1,794) of the municipalities in the Northeast region achieved coverage of 80% or more regarding the first dose for girls, while the percentage of coverage found in the second dose for girls was 15.2% (Figure 3).

When taking into consideration the states, none of them reached 80% homogeneity for any of the doses, and in neither of the sexes. Among boys, 80% homogeneity was found in 12.7% (228/1,794) of the Northeastern municipalities for the first dose of the vaccine. The state of Paraíba contributed the most (28.6%) to the homogeneity indicator among the municipalities that reached this coverage. None of the states reached 80% coverage or more among the male population for both doses (first or second). Regarding the girls, the state with the best result of homogeneity of first-dose coverage was Paraíba, with 76.6% of its municipalities with adequate vaccination coverage. This state also showed the highest proportion (32.7%) of municipalities with coverage greater than or equal to 80% in the second dose. The state of Paraíba also presented the highest homogeneity of coverage for males - 28.6% for the first dose and 9.4% for the second dose (Table 1).

**Table 1:** - Number of doses and percentages of coverage of human papillomavirus (HPV) vaccine, according to sex, in the Northeast region and its states, Brazil, 2013-2021 (N = 11,613,518)

Sex	Vaccination coverage (%)	Alagoas	Bahia	Ceará	Maranhão	Paraíba	Pernambuco	Piauí	Rio Grande do Norte	Sergipe	Total
**Female**	**1^st^ dose**	n (%)									
	< 50	5 (4.9)	43 (10.3)	9 (4.8)	19 (8.7)	3 (1.3)	6 (3.2)	15 (6.6)	12 (7.1)	1 (1.3)	113 (6.2)
	50 to < 80	41 (40.1)	207 (49.6)	65 (35.3)	109 (50.2)	49 (21.9)	85 (45.9)	93 (41.5)	88 (52.6)	36 (48.0)	773 (43.0)
	≥ 80	56 (54.9)	167 (40.0)	110 (59.7)	89 (41.0)	171 (76.6)	94 (50.8)	116 (51.7)	67 (40.1)	38 (50.6)	908 (50.6)
	**2^nd^ dose**										
	< 50	31 (30.3)	153 (36.6)	39 (21.1)	70 (32.2)	21 (9.4)	35 (18.9)	57 (25.4)	65 (38.9)	12 (16.0)	483 (26.9)
	50 to < 80	66 (64.7)	211 (50.5)	111 (60.3)	130 (59.9)	129 (57.8)	124 (67.0)	130 (58.0)	85 (50.8)	52 (69.3)	1.038 (57.8)
	≥ 80	5 (4.9)	53 (12.7)	34 (18.4)	17 (7.8)	73 (32.7)	26 (14.0)	37 (16.5)	17 (10.1)	11 (14.6)	273 (15.2)
**Male**	**1^st^ dose**										
	< 50	56 (54.9)	166 (39.8)	70 (38.0)	94 (43.3)	33 (14.7)	65 (35.1)	70 (31.2)	74 (44.3)	25 (33.3)	653 (36.3)
	50 to < 80	40 (39.2)	207 (49.6)	94 (51.0)	119 (54.8)	126 (56.5)	105 (56.7)	110 (49.1)	72 (43.1)	40 (53.3)	913 (50.8)
	≥ 80	6 (5.8)	44 (10.5)	20 (10.8)	4 (1.8)	64 (28.6)	15 (8.1)	44 (19.6)	21 (12.5)	10 (13.3)	228 (12.7)
	**2^nd^ dose**										
	< 50	95 (93.1)	319 (76.4)	140 (76.0)	191 (88.0)	123 (55.1)	151 (81.6)	151 (67.4)	131 (78.4)	51 (68.0)	1.352 (75.3)
	50 to < 80	6 (5.8)	86 (20.6)	38 (20.6)	25 (11.5)	79 (35.4)	31 (16.7)	67 (29.9)	30 (17.9)	23 (30.6)	385 (21.4)
	≥ 80	1 (0.9)	12 (2.8)	6 (3.2)	1 (0.4)	21 (9.4)	3 (1.6)	6 (2.6)	6 (3.5)	1 (1.3)	57 (3.1)

## DISCUSSION

Between 2013 to 2021, HPV vaccination coverage found in the states in the Northeast region was, in general, below the 80% goal recommended by the PNI. Vaccination coverage was higher for girls and it was higher in the first dose for both sexes. Ceará and Paraíba reached coverage above 80% in the first dose, among girls. No states in the region reached the goal for the other doses.

As a limitation of this study, we point out the potential inaccuracy of the coverage calculation, given that this study was conducted based on secondary data (indirect method), which may present deficiency in attributes such as opportunity, data quality, completeness, among others.^17^ As we are dealing with anonymized secondary data, it was not possible to verify the consistency of the records regarding their correctness, so that eventual registration errors related to sex and age group constitute potential biases.

The number of doses administered may not correspond to the number of people vaccinated, since there may be duplicate records and vaccine loss, and it is possible the occurrence of errors in the record of the order of the dose administered: first or second dose. The denominator is based on an estimated population of people in the age group eligible to receive the vaccine. Because it is an estimate, it is more subject to inaccuracies than a census. People can also be vaccinated outside their municipality of residence, which influences the estimate, and coverage can be increased in one municipality and reduced in another. This limitation can be overcome by studies that use the direct method, with local measurement by means of vaccination coverage surveys - a more accurate method, although it is more expensive and time-consuming.

The low vaccination coverage found in this study, in most states in the Northeast region, points to the existence of a worrying situation, taking into consideration that this region has the highest prevalence of HPV infection, it is the second region with the highest mortality rate of cervical cancer in women in the country,^7^ ^,^ ^18^ ^,^ ^19^ and has in one of its states the highest incidence of penile cancer in the world.^4^ It is noteworthy that the control of immuno-preventable diseases depends, among other factors, on wide vaccination coverage. In the specific case of HPV vaccination, it is postulated that the minimum coverage required is 80%.^14^

Only the states of Ceará and Paraíba reached the goal of vaccination coverage for the first dose among girls. The best coverage in these states may be related to specific legislation and actions that link vaccination to school activities, in addition to conducting periodic campaigns on social media about the importance of HPV vaccination. With regard to an evaluation of HPV vaccine coverage conducted by the Ministry of Health nationwide, with data from 2013 to 2018, two states in the Northeast region (Ceará and Pernambuco) exceeded the goal for the first dose, and five states were below the national average for the second dose.^20^

In the present study, the vaccination coverage presented for males was lower than that for females, in all states and for both doses. In contrast to these results, a descriptive cross-sectional study conducted in 24 municipalities in Paraíba found a higher first-dose vaccination coverage for males.^21^ The study took into consideration data from 2017, related to 18 municipalities in the state, and included doses administered to people aged up to 26 years and with comorbidities, which may explain the differences that were found.

The lowest vaccination coverage among boys may be related to the lack of knowledge of families about the negative impact of HPV on males.^20^ ^,^ ^22^ ^-^ ^24^ The lack of knowledge of the general population about HPV was identified in a cross-sectional study conducted between September 2016 and November 2017, in primary healthcare centers located in the 27 FUs in Brazil, with 2,125 men and 5,569 women who were sexually active, unvaccinated, and their mean age was 21.6 years: the results showed that half of young people aged 16 to 25 year old are still unaware of the association between the HPV and the onset of cancer.^25^ A study conducted in the United Kingdom in 2016 and 2017, with 186 parents of adolescents aged 11 to 18 years, observed that among respondents who had heard about HPV, knowledge about the health sequelae of HPV for men was poor in relation to their knowledge about its impact on female health.^23^ In Brazil, interviews conducted with 826 people in the seven largest cities in the country, between 2015 and 2016 report that among parents who refused HPV vaccine for their sons but accepted it for their daughters, the most common reason for that was “the HPV vaccine is not recommended for boys.”^22^ It is worth mentioning that the efficacy and safety of HPV vaccine in this public has already been demonstrated in a double-blind, randomized, placebo controlled trial, which included 1,803 men aged 16 to 26 years old, in 18 countries.^26^

It is important that vaccination coverage be high and adequately distributed, which was not observed even in states that reached over 80% coverage goal.^14^ In Ceará, for example, although the state presented coverage above the goal in its territory, it could be seen that this coverage occurred in just over half of the municipalities. This means that even if the state has met the goal of vaccination coverage, there may be an uneven distribution among its municipalities, that is, the distribution is not homogeneous. For the Northeast region as a whole, homogeneity was 18%, with 330 of the 1,794 municipalities presenting coverage greater than or equal to 80% for the second dose. The heterogeneity of coverage was observed in an evaluation of vaccination coverage by age cohorts of girls, conducted with data from 2013 to 2017.^27^ No studies were found that evaluated the homogeneity of vaccination coverage among the male population.

Strategies to improve vaccination coverage aimed at adolescents, not only in healthcare centers, but also in extramural contexts, especially those carried out in the school environment,^28^ ^,^ ^29^ can increase adherence to prevention actions among adolescents and promote the improvement of vaccination coverage. A prospective cohort study with 4,878 boys and girls aged 9 and 10 years in the city of Indaiatuba, state of São Paulo, in 2018 and 2019, showed an increase in vaccination coverage from 16% to 50% in the first dose, after the implementation of vaccination strategies in the school environment.^12^

It can be concluded that, in the period from 2013 to 2021, HPV vaccination coverage in girls and boys is below the target in most states in the Northeast region. Studies based on vaccine coverage survey, in addition to plans with specific local vaccination strategies for each state, can be implemented in order to achieve the vaccination coverage target in the region.

It is recommended to intensify communication actions aimed at the population and health professionals, in addition to increasing vaccination in the territory, with actions integrated with other strategies, such as the School Health Program (Programa Saúde na Escola - PSE). It is also recommended to strengthen the actions of the Family Health Teams (Equipes de Saúde da Família - ESF), especially regarding their home visits and community mobilization actions.
